# Supplementary material for: Cold Atmospheric Plasma Increases Temozolomide Sensitivity of Three-Dimensional Glioblastoma Spheroids via Oxidative Stress-Mediated DNA Damage
Source: Cancers (Basel). 2021 Apr 8;13(8):1780. doi: 10.3390/cancers13081780 (PMC8068248; doi:10.3390/cancers13081780)
Supplement: Supplementary file 1 [file cancers-13-01780-s001.pdf]

cancers

Supplementary Materials

Cold atmospheric plasma increases temozolomide sensitivity of three-dimensional glioblastoma spheroids via oxidative stress-mediated DNA damage

Priyanka Shaw[1, 2*], Naresh Kumar[1, 3], Angela Privat-Maldonado[1, 2], Evelien Smits[2], and Annemie Bogaerts[1*]

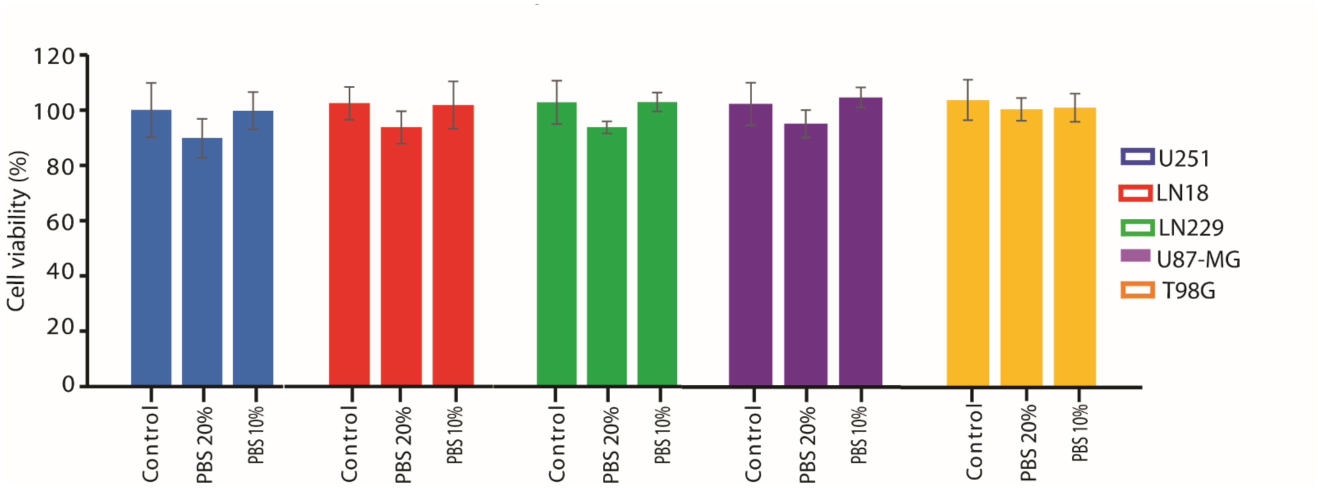

Figure S1. Dose-response survival curves of untreated PBS 20% and PBS 10% after 24 hour of stimulation in U251, LN18, LN229, U87-MG and T98G.

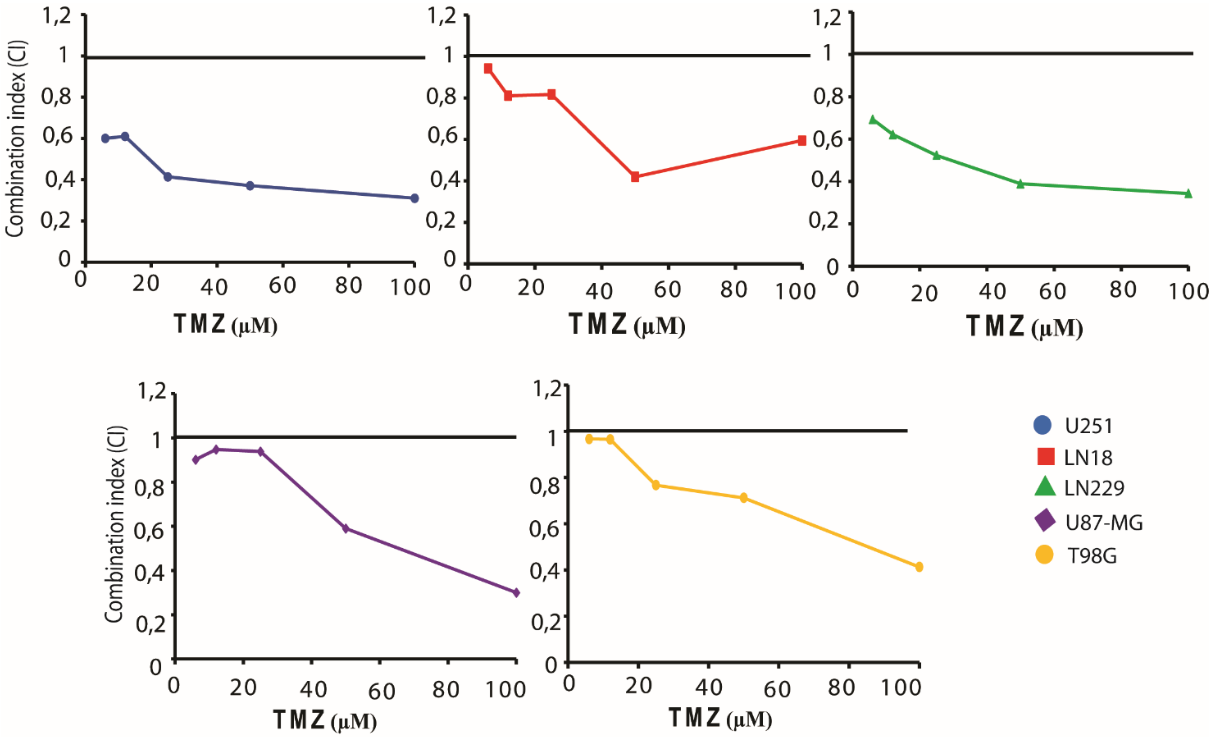

Figure S2. Combination index (CI) of (a) PT-PBS (10%) with TMZ (at 100, 50, 25, 12.5 and 6 µM) in U251, (b) PT-PBS (10%) with TMZ (at 100, 50, 25, 12.5 and 6 µM) in LN18, (c) PT-PBS (10%) with TMZ (at 100, 50, 25, 12.5 and 6 µM) in LN229, (d) PT-PBS (15%) with TMZ (at 100, 50, 25, 12.5 and 6 µM) in U87-MG, and (e) PT-PBS (20%) with TMZ (at 100, 50, 25, 12.5 and 6 µM) in T98G.
